# RE: A Novel method of ensuring safe and accurate dilatation during percutaneous nephrolithotomy

**DOI:** 10.1590/S1677-5538.IBJU.2016.0017

**Published:** 2016

**Authors:** Tarun Javali, Amey Pathade, H. K. Nagaraj

**Affiliations:** 1 M.S. Ramaiah Hospital, Bangalore, India

*To the editor,*

We have read the technique titled “A novel method of ensuring safe and accurate dilatation during percutaneous nephrolithotomy” by Javali et al (1) with great interest. We compliment the authors on this novel technique of placing guide wire in difficult situation.

We would like to draw the attention of the authors to a few points and make a few comments.

A well-placed guide wire is a corner stone to the success of percutaneous access. Failure of guide-wire access may result in potential complications such as loss of tract, bleeding due to parenchymal injury that might lead to abandonment of procedure (2). Although wire down the ureter in every case would be ideal, but it is not necessary to be very rigid about that. We feel adequate and secure length of the wire in the pelvi-calyceal system is all that is needed for a satisfactory and safe tract making.

The common causes of guide-wire not going up the renal pelvis or down the ureter (3) are:

- Large calculus occupying and/or blocking the calyx or infundibulum,
- Puncture of the anterior calyx instead of the posterior calyx.

If a large obstructing calculus is the cause of wire not progressing, there may be a need to fragment the calculus to create space for the wire to proceed. The authors have not mentioned any incidence of need for fragmentation in this large series.

The lower pole calyces usually have a complex arrangement. The typical anterior and posterior arrangement of calyces is seen only in 58% of cases in the lower pole (3). Eisner et al have found that in 31% of cases, the arrangement of calyces in the lower pole is such that no calyx is truly posterior. In such kidneys both the calices in the lower pole are anterior with one of the calyx being less anterior as compared to the other (4). If the anterior calyx is punctured then the glide wire would have difficulty in entering the pelvis (2). In this situation, gaining access to renal pelvis and upper ureter using an ureteroscope would torque the lower pole. This can be traumatic.

In a lower calyx tract (51 out of 85) it would be very difficult even under ureteroscopic vision to make the wire go down the ureter. The angle between the lower calyx infundibulum and the upper ureter would make entry of the wire across pelvi-ureteric junction difficult (5). In this situation placing the wire in upper calyx would be far easier.

The authors have passed a 6-7.5 Fr semi-rigid ureteroscope between two terumo guide-wires and have managed to keep the two guide-wires always in vision. This would be very difficult in patients with prior history of renal surgery (12 in this series). Posterior strong muscular support with retractile property of muscles would increase this difficult. There is a chance of perforation of the calyceal system especially with semi-rigid ureteroscope. There are reports of use of flexible ureteroscopes (6, 7) for antegrade ureteroscopic assistance in percutaneous nephrolithotomy to prevent these potential damages.

This technique needs use of three guide-wires in addition to the use of the ureteroscope. There is a potential for damage to the optics of the telescope especially in patients with large stone burden with impacted calculi. All these aspects would increase the cost of the procedure. Is this escalated cost really necessary and justified?

*Pankaj N. Maheshwari, MS, DNB, MCh (Urology)* *Senior Consultant* & *Chief of Urology* *Fortis Hospital Mulund* *Mulund-Goregoan Link Road* *Mulund-West* *Mumbai 400078, India* *Fax:+91 22 6799-4242* *Email: dr.maheshwaripn@gmail.com* *Gyanendra R. Sharma, MS, MCh (Urology)* *Chitale Urology center, Solapur, Maharashtra, India* *E-mail: drgrsharma@gmail.com* *Vinayak G. Wagaskar, MS* *King Edward Memorial Hospital, Mumbai, India* *E-mail: vinayakwagaskar99@gmail.com*

## References

[1] Javali T, Pathade A, Nagaraj HK (2015). A Novel method of ensuring safe and accurate dilatation during percutaneous nephrolithotomy. Int Braz J Urol.

[2] Sharma GR, Maheshwari PN, Sharma AG, Maheshwari RP, Heda RS, Maheshwari SP (2015). Fluoroscopy guided percutaneous renal access in prone position. World J Clin Cases.

[3] Sampaio FJ (2000). Renal anatomy. Endourologic considerations. Urol Clin North Am.

[4] Eisner BH, Cloyd J, Stoller ML (2009). Lower-pole fluoroscopy-guided percutaneous renal access: which calix is posterior?. J Endourol.

[5] Maheshwari PN, Oswal AT, Andankar M, Nanjappa KM, Bansal M (1999). Is antegrade ureteroscopy better than retrograde ureteroscopy for impacted large upper ureteral calculi?. J Endourol.

[6] Tsai YS, Jou YC, Shen CH, Lin CT, Chen PC, Cheng MC (2015). Antegrade ureteroscopic assistance during percutaneous nephrolithotomy for complete renal staghorn stone: Technique and outcomes. Urological Science.

[7] Kawahara T, Ito H, Terao H, Yoshida M, Ogawa T, Uemura H (2012). Ureteroscopy assisted retrograde nephrostomy: a new technique for percutaneous nephrolithotomy (PCNL). BJU Int.

